# Methods to Estimate Acclimatization to Urban Heat Island Effects on Heat- and Cold-Related Mortality

**DOI:** 10.1289/ehp.1510109

**Published:** 2016-02-09

**Authors:** Ai Milojevic, Ben G. Armstrong, Antonio Gasparrini, Sylvia I. Bohnenstengel, Benjamin Barratt, Paul Wilkinson

**Affiliations:** 1 Department of Social and Environmental Health Research, London School of Hygiene & Tropical Medicine, London, United Kingdom; 2 Department of Meteorology, University of Reading, Reading, United Kingdom; 3 Environmental Research Group, King’s College London, London, United Kingdom

## Abstract

**Background:**

Investigators have examined whether heat mortality risk is increased in neighborhoods subject to the urban heat island (UHI) effect but have not identified degrees of difference in susceptibility to heat and cold between cool and hot areas, which we call acclimatization to the UHI.

**Objectives:**

We developed methods to examine and quantify the degree of acclimatization to heat- and cold-related mortality in relation to UHI anomalies and applied these methods to London, UK.

**Methods:**

Case–crossover analyses were undertaken on 1993–2006 mortality data from London UHI decile groups defined by anomalies from the London average of modeled air temperature at a 1-km grid resolution. We estimated how UHI anomalies modified excess mortality on cold and hot days for London overall and displaced a fixed-shape temperature-mortality function (“shifted spline” model). We also compared the observed associations with those expected under no or full acclimatization to the UHI.

**Results:**

The relative risk of death on hot versus normal days differed very little across UHI decile groups. A 1°C UHI anomaly multiplied the risk of heat death by 1.004 (95% CI: 0.950, 1.061) (interaction rate ratio) compared with the expected value of 1.070 (1.057, 1.082) if there were no acclimatization. The corresponding UHI interaction for cold was 1.020 (0.979, 1.063) versus 1.030 (1.026, 1.034) (actual versus expected under no acclimatization, respectively). Fitted splines for heat shifted little across UHI decile groups, again suggesting acclimatization. For cold, the splines shifted somewhat in the direction of no acclimatization, but did not exclude acclimatization.

**Conclusions:**

We have proposed two analytical methods for estimating the degree of acclimatization to the heat- and cold-related mortality burdens associated with UHIs. The results for London suggest relatively complete acclimatization to the UHI effect on summer heat–related mortality, but less clear evidence for cold–related mortality.

**Citation:**

Milojevic A, Armstrong BG, Gasparrini A, Bohnenstengel SI, Barratt B, Wilkinson P. 2016. Methods to estimate acclimatization to urban heat island effects on heat- and cold-related mortality. Environ Health Perspect 124:1016–1022; http://dx.doi.org/10.1289/ehp.1510109

## Introduction

It is well known that urban areas can experience ambient temperatures appreciably warmer than surrounding rural areas—a phenomenon known as the urban heat island (UHI) effect (Oke 1982). The primary cause is the built environment, which absorbs and stores more heat than natural landscapes; waste heat generated by energy processes in buildings, transport systems, and industry is a second, typically less important, factor in the United Kingdom (Bohnenstengel et al. 2011, 2014). The situation might be different in south-east Asian or U.S. cities. Such variation of ambient temperature can also be observed within a city (warmer inner city and cooler outer city). The UHI effect is typically larger at night than it is during the day (Bohnenstengel et al. 2011; Wilby et al. 2011). From a health perspective, the additional summer heat of the UHI is of concern because of its potential exacerbation of heat-related health risks, which, in many settings, are projected to worsen as a consequence of climate change (Hajat et al. 2014; Vardoulakis et al. 2014). Many city authorities are actively considering how the UHI effect may be minimized by improved land-use planning, additional tree planting, and other interventions. However, there is only limited direct empirical evidence on the magnitude of the UHI risks to health.

In this study, our primary focus concerned the UHI effects operating within a city. Relatively few studies have explored intra-city variation in heat-related mortality (Gabriel and Endlicher 2011; Goggins et al. 2013; Harlan et al. 2013; Reid et al. 2009; Smargiassi et al. 2009; Vandentorren et al. 2006; Xu et al. 2013), which may arise not only because of the UHI effect (Harlan et al. 2013) but also because of variations in the vulnerability of the population from such factors as population age or socioeconomic deprivation (Reid et al. 2009; Xu et al. 2013). A study in Montreal (Smargiassi et al. 2009) found a greater risk of death on hot summer days in areas with high surface temperatures as defined by satellite images, and a German study (Gabriel and Endlicher 2011) found a positive correlation between excess mortality during periods of high heat stress and the proportion of land area covered by sealed surfaces in a district. A case–control study of deaths among an elderly population during the 2003 heat wave in France (Vandentorren et al. 2006) reported an increased risk of all-cause death in areas with a 1°C higher surface temperature index, which was generated from satellite thermal infrared images [adjusted odds ratio of 1.82; 95% confidence interval (CI): 1.27, 2.60].

Studies of UHI effects have mainly been limited to analyses of heat effects, with very little focus on possible attenuation of cold effects. For instance, inner-city areas may experience fewer cold-related deaths than outer-city areas because of the UHI effect. Few studies have attempted to separate UHI influences from other sources of variation in population vulnerability such as socioeconomic deprivation (Goggins et al. 2012) or population age. Moreover, to our knowledge, no studies have clarified whether the size of the UHI-related excess of heat mortality was commensurate with the extent of the difference in temperature. Although multicity studies showed some evidence of possible adaptation or acclimatization to the local climate—hotter cities often did not experience as much of an increase in heat-related mortality over cooler cities as might have been expected from the difference in temperature (Curriero et al. 2002)—it is not known whether parts of cities experiencing more heat as a result of the UHI effect showed any such decreased susceptibility.

In this study, we present methods to determine whether hotter neighborhoods (those affected by a UHI) have higher excess mortality on hot days (or lower mortality on cold days), allowing for adjustment of other factors, and to estimate the extent to which such differences are consistent with expectations given how much hotter or colder those areas are compared with London overall. For brevity, we refer to apparent differences in susceptibility to the effects of heat or cold among UHI-anomaly decile groups as evidence for or against local acclimatization to the UHI effect. Here, we refer to a difference in susceptibility among neighborhoods rather than to a change in susceptibility over time within a single population. The underlying causes are unknown and may include physical components such as built environment and physiological mechanisms, whether such changes are consciously made to adapt or not [more restrictive uses are reported in Gosling et al. (2014) and in IPCC (2014)]. In this paper, we present the above-mentioned methods and apply them to data from London in the period 1993–2006, and we consider modification of both cold and heat effects by the UHI effect.

## Methods

### Data

The present study was based on an analysis of daily mortality for all-cause deaths in London, 1993–2006, with individual mortality records [Office for National Statistics (ONS) 2004] linked to the area of residence through the address postal code [on average, 18 households or 43 residents per residential postal code in England (ONS 2004)]. A single London series of temperature for the same period was constructed as the population-weighted average of the daily mean temperatures at seven available monitoring sites, imputing missing values by the method of the AIRGENE study (Rückerl et al. 2007); details are available (Armstrong et al. 2011).

In the present study, UHI was considered as a primary modifier of main temperature effect on mortality. Socioeconomic deprivation could also be a possible effect modifier of the temperature–mortality relationship, which might confound UHI effects (as an effect modifier) on the temperature–mortality relationship (details below). As such, we assembled data from the English Index of Multiple Deprivation (EIMD) 2004 for the lower layer super output area (LSOA) of residence (Office of the Deputy Prime Minister 2004). The LSOA is a unit of small area that is designed to be homogeneous in neighborhood characteristics and has a relatively even population size of 1,500 residents on average. The EIMD 2004 was modified by excluding two domains (the health and disability domains and the living environment domain) that partially included variables to be incorporated in the main analytical model (small-areal statistics of mortality and ambient concentration of particulate matter and other air pollutants, respectively), keeping the overall weights of the five remaining domains (income; employment; education, skills, and training; barriers to housing and services; crime) proportional to those in the original index, following the approaches used in previous studies (Adams and White 2006; Goodman et al. 2011).

Single London series of air pollution levels for the daily mean PM_10_ (particulate matter with aerodynamic diameter < 10 μm) and the daily maximum of 8-hr running mean ozone (O_3_) in 1993–2006 were also constructed from urban background and suburban monitoring sites located in greater London (35 sites, 18 nonmissing measures on average per day for PM_10_; 29 sites, 15 nonmissing measures for O_3_). Pollution measurements were obtained from the London Air Quality Network managed by King’s College London (http://www.londonair.org.uk). Geographical data linkages were conducted in ArcGIS v.10.0.

### Modeling the UHI

**Figure 1:**
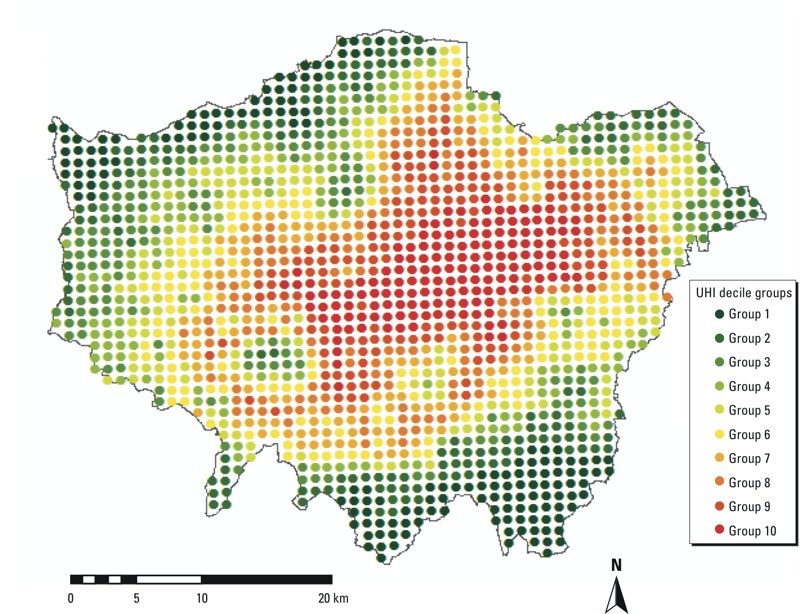
London urban heat island (UHI) anomaly decile groups. UHI anomalies were defined by the annual mean of daily excess temperature at each grid square relative to the average temperature on the same day in London as a whole. Decile group 1 represents the lowest UHI anomaly group (coolest), and decile group 10 represents the highest UHI anomaly group (hottest).

In order to quantify the UHI, modeled ambient temperatures in London (degrees Celsius) at a height of 1.5 m were derived at 1-km grid resolution from numerical simulations using the Met Office weather forecast model (Unified Model). Within the Unified Model, a parameterization for urban land-use was used to calculate the exchange of heat, momentum, and moisture between the urban land surface (i.e., street canyons) and the atmosphere. The Met Office Reading Surface Exchange Scheme (MORUSES) was used to calculate the surface energy balance, that is, the sensible heat flux, the storage of heat in the buildings and the ground, and long-wave and short-wave radiation based on the geometry of street canyons. Details about the MORUSES parameterization are available elsewhere (Bohnenstengel et al. 2011). For each day and each grid square, the excess temperature relative to the London mean for that day was calculated, and the daily excesses were averaged over all days in the available model data (May to August and December 2006). This variable is called the annual urban heat island anomaly (UHIa), and that at grid square *g* is expressed as:


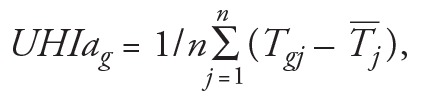


where *T_gj_* is the maximum temperature at grid square *g* on day *j*, *T_j_* is the average daily mean temperature across all grids in London on day *j*, and *n* is the number of days (here, *n* = 154). All 1-km grids (1,587 grids in London) were classified into decile groups based on the decile of distribution of grid UHI anomalies (UHIas) in London. Figure 1 presents the spatial distribution of these UHI anomaly decile groups. Table 1 summarizes the averaged UHIa for each UHI decile and the corresponding statistics.

**Table 1:** UHI anomaly, deprivation index*,* and all-cause deaths for London UHI anomaly decile groups.

UHI decile groups^*a*^	Mean UHIa^*b*^ (°C)	Mean deprivation index^*c*^ (*z*-score)	Number of all-cause deaths	Percent of ≥ 75 years old deaths
Group 1	–0.93	–0.62	23,170	66.7
Group 2	–0.51	–0.41	44,007	67.5
Group 3	–0.26	–0.41	63,721	66.5
Group 4	–0.11	–0.28	76,293	64.3
Group 5	0.01	–0.17	83,281	63.0
Group 6	0.12	–0.33	87,214	62.1
Group 7	0.23	–0.03	99,339	61.5
Group 8	0.34	0.33	103,658	60.5
Group 9	0.47	0.78	130,458	55.4
Group 10	0.63	1.18	132,396	52.7
Abbreviations: UHI, urban heat island; UHIa(s), urban heat island anomaly (anomalies). ^***a***^UHI decile groups were defined by the deciles of all grid UHIas in London. Group 1 represents the smallest UHIa group, and Group 10 represents the largest UHIa group. ^***b***^UHIa is the annual average of the daily excess temperature at each grid square relative to the average temperature on the same day in London as a whole. ^***c***^Deprivation index was reconstructed from the English Index of Multiple Deprivation 2004 [Office of the Deputy Prime Minister (ODPM) 2004], excluding the health and disability domains and the living environment domain.				

### Statistical Methods

Analysis of the relationship between mortality risk and daily mean temperature was based on a case–crossover analysis stratified by year, month, and UHIa decile groups, using a conditional Poisson model (Armstrong et al. 2014). This can be equivalently thought of in case–control study terms as case–control sets, each comprising explanatory variable values for 1 case day (if there was a death that day) and 27–30 control days (same calendar year, month, and UHI decile group). All analyses controlled for day of the week and for count of circulating influenza (from the Communicable Diseases Surveillance Centre) by including these as explanatory variables.

Algebraically, the formula can be written as follows:

*Y_ij_ ~ Poisson (μ_ij_ | total deaths in UHI group i, year and month) with μ_ij_ = exp{(covariates) + (terms involving temperature t_j_ and UHIa_i_)},* [1]

where*Y_ij_* is the death count on day *j* and UHIa decile group *i;* covariates are the linear sum of regression terms (coefficient × variable), Σ(β*_k_* × *Z_kj_*), for deaths from influenza in England and Wales on day *j* and indicator terms for days of the week; *t_j_* is the mean ambient temperature on average over all London on day *j*; and UHIa*_i_* is the mean UHIa anomaly (in degrees difference to London mean) in UHIa group *i*.

The main effect of temperature on mortality was modeled separately for summer (June–August) and winter (September–May) with distributed lag nonlinear models using the dlnm R package (Gasparrini 2011) with unconstrained lags 0–1 (same day and day before) for summer and a natural cubic spline lag structure with two knots (package default placement) over lags 0–13 for winter. The lag intervals were chosen based on previous work (Hajat et al. 2007). We used two approaches to model the impact of UHI on temperature effects: a crude appraoch similar to methods that have been used previously (Goggins et al. 2013; Smargiassi et al. 2009; Vandentorren et al. 2006) and a more sophisticated but possibly less transparent one.

***Comparison of the risk for deaths on hot and cold days (relative to that on days with moderate temperatures) at UHIa of +0.5 and –0.5°C.*** For this analysis, the heat and cold risks were modeled (separately for each season) as simple dichotomies: indicators for “hot” and “cold” days:

*μ_ij_ = exp{(covariates) + dlnmA(t_j_)}* [2]

where *dlnmA* is a *dlnm* with temperature dichotomy (hot or cold day) and lag structure as described for model [1].

Cut-points used to define hot and cold day indicators were 22.3 and 6.4°C, respectively, chosen as the temperatures that gave the most significant risk excesses, measured by the Wald *z* = log(RR)/SE(log(RR) over a range of trial values (see Figure S1).

We modeled the UHIa modification of these heat- and cold-related mortality risks as interaction (product) terms for each dlnm sub-term:

*μ_ij_ = exp{(covariates) + dlnmA(t_j_) +* θ × *UHIa_i_* × *dlnmA(t_j_)}.* [3]

We present the results from the fitted models as the relative change in these predicted heat (cold) mortality ratios for a UHIa of +0.5°C compared with that for a UHIa of –0.5°C (one degree difference). We refer to this relative change associated with one degree UHIa as the interaction rate ratio (IRR). One degree of UHI anomaly is slightly less than the difference in the mean anomaly between the lowest and the highest UHIa decile group (–0.93 and 0.63°C, respectively; range 1.56).

These IRRs estimate the increased risk on hot days in areas of London subject to the UHI compared with areas typically one degree cooler by the UHIa (and analogously for cold). We sought to compare these estimates with what would be expected from the overall increased risk in London for days that are one degree hotter (colder). To perform this comparison, we estimated the heat (cold) slope of the mortality increment in association with the London-wide daily mean temperature, ignoring the modification by UHI of the temperature–mortality relationship. This model was the same as model [2] above but fitted the temperature effect as a linear spline (segmented linear model) with knots at 18.6 [the minimum mortality temperature (MMT) in a natural cubic spline all-year model] and 22.3°C for heat (see Figure S2), and 6.4 and 18.6°C for cold. The expected IRR for heat was estimated as the slope in the spline above the highest knot (below the lowest for cold). IRRs for heat at the expected value indicate no acclimatization to heat in a UHI, and IRRs below that value indicate a degree of such acclimatization (reduced vulnerability).

***Comparison of the displacement, parallel to the temperature axis, of a fixed-shape temperature–mortality function at UHIas of +0.5 and –0.5°C.*** The second method entailed fitting a temperature–mortality curve for each season (natural cubic splines) and quantifying the displacement of this function parallel to the temperature axis at different UHIas under the constraint that the function has identical shape at all UHIas and is displaced linearly with the UHIa. Algebraically, this expression can be written as follows:

*μ_ij_ = exp{(covariates) + dlnmB(t_j_ +* γ*UHIa_i_)}* [4]

With *dlnmB*(*t*) having a natural cubic spline temperature function ncs with 4 df (chosen *a priori* by experience).

The extent to which the curve was displaced by the UHIa (γ) was estimated by calculating likelihoods (deviances) over a grid of candidate values and thereby obtaining the maximum likelihood estimate. We refer to this as the “shifted spline” method. As with the first method, although UHIa was again fitted as a continuous variable, we report the extent to which the splines were shifted from a UHIa of +0.5°C to a UHIa of –0.5°C.

The results of the “shifted spline” analysis are shown in terms of the displacement parameter, γ, which, for heat, represents the displacement of the temperature–mortality function for one degree UHIa, for example at UHIa of +0.5°C compared with that at UHIa of –0.5°C. If there is no acclimatization, γ takes the value 1, indicating that the observed curves (at UHIa +0.5 and –0.5°C) are separated by the actual temperature differences between those areas, namely, 1°C in this instance (see Figure S3A). Under full acclimatization, γ takes the value 0 and the curves at UHIa = +0.5 and UHIa = –0.5°C will be superimposed because the population exhibits the same temperature–mortality function (shape and location with respect to the single temperature series) in all areas (see Figure S3B). The same interpretation applies for cold-related mortality but with comparison of the curves at UHIa reversed: –0.5 versus +0.5°C.

Differences between deviances at the fitted value for γ = 0 and γ = 1 provide likelihood ratio tests against null hypotheses of full and no acclimatization, respectively.

Key to the interpretation of both measures of effect modification by the UHIa is that, in our analyses, the temperature-mortality relationship was based on a single “average” temperature series for London. This assumption means that the actual temperature experienced by the population at grid locations with positive values for UHIa is underestimated by the single series, whereas those with negative values for UHIa is overestimated. Thus, if the true temperature–mortality relationship is identical in all locations of London, regardless of the UHIa (we call this full acclimatization), then we would expect higher relative risks for heat in areas with a positive UHIa because the actual temperatures are higher than those indicated by the single temperature. Similarly, there would be lower relative risks for heat in areas with a negative UHIa because the actual temperatures would be lower than those indicated by the single series.

### Control for Other Possible Biases of the UHI Effect

Although age and socioeconomic deprivation are time-invariant in the context of this analysis, and therefore are not potential confounders in the usual sense, they both could confound the estimated modification of heat (cold)-mortality associations (IRRs) by UHI if they also modified those associations. We controlled for this possibility in additional analyses. For socioeconomic deprivation, we entered an average of reconstructed EIMD scores by UHI decile groups into the simpler model (the first method) as a second modifier of heat and cold (i.e., further interaction terms in model 3). We also checked whether socioeconomic deprivation actually modified the heat- and cold-related mortality associations as a first modifier. For age, because of its stronger expected modification of heat and cold risks, we instead stratified our main analyses by age groups (0–64, 65–74, ≥ 75 years), particularly focusing on elderly people, who are known to have increased vulnerability to the effects of heat and cold. Ambient pollution (O_3_ and PM_10_) is a time-varying risk factor, so we adjusted for their effects by directly including them in the model as linear terms, although we note that this adjustment might be better considered as controlling for indirect temperature effects mediated through O_3_ and PM_10_ than as simply controlling confounding (Buckley et al. 2014).

As a sensitivity analysis, we repeated the main analyses with shortened non-summer months (October–April) to reduce possible confounding by heat in September and May. All confidence intervals (CIs) shown in the results represent 95% CIs. Statistical analyses were performed in R v.3.0.2 (R Core Team 2013); R code is available for individual request to the first author.

## Results

### Hot and Cold Versus Moderate Temperature Periods

The results of the comparison of mortality risks in the hot and cold temperature ranges relative to that in the moderate temperature range are shown in Table 2. In the unadjusted analysis, the point estimate of the heat-related mortality risk at the UHIa of +0.5°C was 1.208 (95% CI: 1.176, 1.241), slightly higher than the value of 1.203 (95% CI: 1.154, 1.255) obtained at the UHIa of –0.5°C. The confidence interval for the IRR was compatible with no difference (1.004, 95% CI: 0.950, 1.061). This IRR compares with an expected ratio of 1.070 (95% CI: 1.057, 1.082) if no acclimatization is assumed; that is, if areas at different UHIas have the same level of risk in relation to the actual temperatures experienced in those areas. Thus, the observed results suggest only small differences in heat risk between areas with anomalies at +0.5°C and –0.5°C compared with the expected IRR assuming no acclimatization, a finding that is most compatible with a fairly high degree of acclimatization to heat. In this situation, the heat-related relative risks in relation to the single temperature series are similar in all areas irrespective of the UHIa.

**Table 2:** Heat- and cold-related RRs at UHIas of +0.5 and –0.5°C, observed IRRs, and IRRs expected in the absence of acclimatization.

Exposure	UHIa^*a*^ (°C)	RR^*b*^ (95% CI)	IRR^*c*^ (95% CI)	Expected IRR (95% CI) assuming no acclimatization^*d*^
Heat	–0.5	1.203 (1.154, 1.255)	1	1
	+0.5	1.208 (1.176, 1.241)	1.004 (0.950, 1.061)	1.070 (1.057, 1.082)
Cold	+0.5	1.129 (1.106, 1.152)	1	1
	–0.5	1.152 (1.116, 1.189)	1.020 (0.979, 1.063)	1.030 (1.026, 1.034)
Abbreviations: IRR, interaction rate ratio; RR, relative risk; UHIa(s), urban heat island anomaly (anomalies). ^***a***^UHIa is the average of excess daily mean temperature (degrees Celsius) at a 1-km grid square compared with the London overall temperature. ^***b***^RRs of mortality for hot and cold days with daily mean temperatures > 22.3°C or < 6.4°C, respectively, compared with days with daily mean temperatures ≥ 6.4 and ≤ 22.3°C, with lag0–1 or lag0–13, respectively, and adjustment for the day of the week and for influenza count. ^***c***^Ratios of the RR for heat in UHIa +0.5 versus –0.5°C, or of the RR for cold in UHIa –0.5 versus 0.5°C. ^***d***^Expected IRRs are generated by modeling the association between mortality and daily mean temperature for London as a whole using a linear spline with knots at 18.6°C (the minimum mortality temperature) and at 22.3°C (for heat) or at 6.4°C and 18.6°C (for cold), with each IRR representing the risk of mortality with a 1°C increase in daily mean temperature > 22.3°C or < 6.4°C for heat and cold, respectively.				

The point estimate results for cold-related mortality suggested a larger relative difference between areas with a UHIa of –0.5°C compared with those with a UHIa of +0.5°C in the unadjusted analyses (IRR = 1.020, 95% CI: 0.979, 1.063), but the confidence interval was compatible with no difference. This figure compares with an expected IRR for cold mortality (if no acclimatization is assumed) in UHIa = –0.5 versus UHIa = +0.5°C of 1.030 (95% CI: 1.026, 1.034). Although the point estimate of the observed IRR (1.02) suggested weak evidence against acclimatization to UHI cold, its wider confidence interval and the relatively small expected IRR (1.030 for cold compared with 1.070 for heat) means that the result is compatible with both no and full acclimatization.

### “Shifted Splines” Analysis

**Figure 2:**
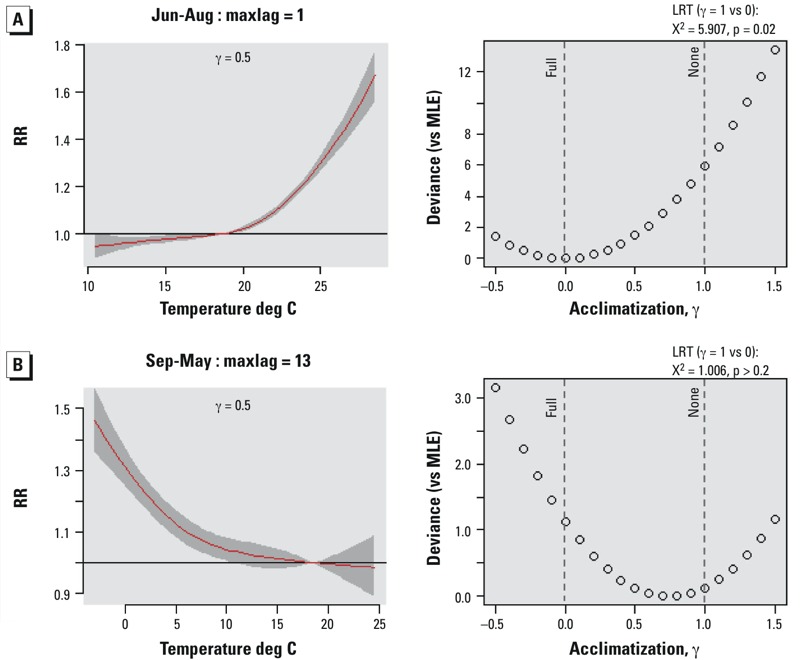
Temperature-mortality functions assuming acclimatization is neutral (γ = 0.5) between full (γ = 0) and none (γ = 1) (left) and deviances of lateral displacement for values of γ in the range –0.5 to 1.5°C (right) for summer heat (lags 0 to 1 days, June to August) (*A*) and winter cold (lags 0 to 13 days, September to May) (*B*). Gray shading in the temperature mortality functions represents the 95% confidence interval. Deviances were calculated against that for the maximum likelihood estimate (MLE). Likelihood ratio test (LRT) was applied for differences between deviances at γ = 1 and γ = 0.

The point estimate of γ for the actual displacement we observed for the high temperature–mortality function in summer was 0 (Figure 2A). Comparison of the deviances indicated that the results were compatible with full acclimatization to heat but not compatible with no acclimatization (*p* = 0.02 by likelihood ratio test). For the low temperature–mortality relationship, the point estimate of γ was 0.8, and therefore was close to that expected with no acclimatization (Figure 2B). However, deviances (i.e., likelihoods) varied little across the range between full and no acclimatization (γ = 0 to 1), indicating that the data were compatible with both hypotheses, and neither hypothesis of full nor no acclimatization to UHI cold would be rejected in a likelihood ratio test.

### Control for Other Possible Biases of the UHI Effect

Little change was observed in heat- or cold-related mortality risk and IRR at different UHIas (the first effect modifier of temperature–mortality relationship) after adjusting for socioeconomic deprivation (an additional potential modifier of temperature effects), although the point estimates for both became marginally < 1 with wider confidence intervals (see Table S1). When we looked at socioeconomic deprivation as a modifier of interest, socioeconomic deprivation itself did not show statistically significant modification of the effects of heat or cold on mortality (unadjusted IRR 1.010, 95% CI: 0.949, 1.074 for heat; and IRR 1.02, 95% CI: 0.980, 1.076 for cold), although the wide confidence intervals did not rule out the possibility of modification (see Table S2). Stratification by age groups did not show much difference in IRR from those overall, although heat- and cold-related relative risks were highest in the ≥ 75 years age group (see Table S3; *p*-values for Cochran’s Q test of heterogeneity, 0.996 for heat and 0.811 for cold). In the “shifted splines” analyses of mortality among the elderly only, the point estimate of γ was 0.3 for both the low and high temperature–mortality relationships, which attenuated the evidence against no acclimatization to UHI heat (*p* = 0.16 vs. *p* = 0.02 for all ages; see Figure S4).

After adjusting for O_3_ and PM_10_, the relative risks for heat were slightly lower in both hotter and cooler areas; thus, there was little change in the IRR itself (1.004, 95% CI: 0.950, 1.061), which remained in conflict with a slightly diminished expected IRR under the no acclimatization assumption (1.059, 95% CI: 1.046, 1.073) (see Table S4). In the “shifted splines” analyses with adjustments for O_3_ and PM_10_, the point estimate of γ for the high temperature–mortality relationship remained close to full acclimatization (γ = 0), and comparison of the deviances showed robust evidence against no acclimatization (*p* = 0.03 by the likelihood ratio test) (see Figure S5). The estimate of the acclimatization parameter, γ, for the low temperature–mortality relationship diminished after adjusting for O_3_ and PM_10_.

Finally, a sensitivity analysis with shortened non-summer months (October–April) showed little difference in the results (see Table S5). Accordingly, the overall findings remained indicative of acclimatization to UHI heat and compatible with both no and full acclimatization to UHI cold.

## Discussion

### Summary of Findings

In this paper, we described a formal approach for quantifying the degree to which populations within the same city are acclimatized to exposure to the higher outdoor temperatures that arise from the UHI effect. We presented two methods: one based on simple comparison of the heat- (cold-) related relative risk at different UHIas and another based on assessment of the degree of lateral displacement (parallel to the temperature axis) at different UHIas of a temperature–mortality relationship constrained to be fixed in shape. With the latter method, in cases where there is no acclimatization, the estimated displacement should exactly match the UHIa. Where there is full acclimatization, the temperature–mortality relationships for all areas (based on an analysis that uses the same single “city average” temperature series) should exactly coincide, such that the actual temperature–mortality functions have altered to such a degree that the mortality risk in the presence of the UHIa on any day is the same as that on the same day in areas with zero anomaly. The proposed methods compared heat- and cold-related mortality among areas with different UHI anomalies (specifically hotter and colder areas) rather than over time, which we used as an indirect method to assess acclimatization to UHI. Application of these methods to London provides some evidence that areas of London subject to UHI-related elevated temperatures in summer have largely acclimatized to these elevated temperatures because both the simple and the “shifted splines” analyses suggested that heat risk depended on London-wide average temperatures and was not increased in areas where the actual local temperatures were higher. However, the evidence was somewhat mixed with regard to cold risk. Before adjustment for socioeconomic deprivation, the results appeared to indicate a situation in which cold risk was reduced where the actual local temperatures were higher (i.e., little acclimatization), but this was not the case after adjustment. Both adjusted and unadjusted results were compatible with full and no acclimatization.

If the lack of increased heat risk in localities with high UHIa indeed reflects acclimatization such as that observed as “adaptation” over long periods of time in Petkova et al. (2014), these findings have relevance to future high-temperature risks under climate change, but it is questionable whether populations would adapt as completely to the rapid and potentially more extreme temperature increments that may result from global warming.

### Strengths and Limitations

The analyses we have presented have a number of strengths and weaknesses. Among the strengths are the comparative richness of the data, with fine geographical coding of death records and detailed socioeconomic and other data available at small area levels, together with the size of the London population, which aids precision because of the comparatively high number of deaths per day. However, even though a sophisticated model was used for assessing temperature variations across London, the UHIa was based on an analysis of a relatively short time period (4 summer months and 1 winter month in 1 specific year at the end of our 14-year mortality series) owing to the limited resources of this project. The use of the annual average UHIa as a marker of the UHI effect can also be debated. However, separate summer and winter UHIas were calculated for each grid in exploratory analyses (with methods identical to those used for all-year calculations) and were found to be highly correlated with the annual average UHIas in this study period. This finding suggests that increasing the amount of UHIa estimates from this study period in London would not change the results very much.

In addition, we controlled only for limited potential biases of the UHI effect, namely, for socioeconomic deprivation, age structure of the population, and selected air pollution levels. Our point estimates of UHI IRRs were robust to adjustment for socioeconomic deprivation except for reducing precision, but because deprivation has not been found to be related to heat mortality risk in London (Ishigami et al. 2008) or to either heat or cold mortality risk in urban areas in England and Wales (Hajat et al. 2007), such adjustment is arguably unnecessary. A possible reason for our observation of little association between heat- and cold-related mortality and socioeconomic deprivation in the United Kingdom could be the generally low prevalence of air-conditioning usage; thus, socioeconomic disparities mediated through air-conditioning use may not be apparent in the United Kingdom, unlike in cities in the United States (Madrigano et al. 2015). We did not attempt more detailed assessment of other variations in the population such as ethnicity (although it is somewhat related to socioeconomic status) or infrastructure by UHI decile groups.

It is generally known that changes in daily ambient temperature influence local air pollution levels, such as the elevation of ozone levels by high temperature through effects on reaction kinetics (Reid et al. 2012) and the possible influence of increased energy consumption on the chemical composition of particulate matter (Anderson et al. 2012). Our main results reflected the total effects of temperature on mortality without controlling for mediation through such air pollution levels. A sensitivity analysis, however, confirmed that the observed relationships were robust to control for indirect mediated effects of temperature through O_3_ and PM_10_.

Another limitation was that the UHIas only reflected outdoor temperature differences at 1.5 m above the land surface. This temperature may be appreciably different from the actual temperature exposures experienced when considering indoor exposures (Barnett 2015), which may be modified by building characteristics (Mavrogianni et al. 2012) and by overshadowing by taller buildings in the city center.

The analytical methods we have proposed are, we believe, the first to quantify the extent to which modification of response to heat (or cold) in UHIas corresponds to that expected with both no and full acclimatization. These methods can be considered to provide formal quantification of the degree of acclimatization to UHIas, the first cost of which was complexity in interpretation, even for the simple “hot and cold periods” analysis. The second cost (limitation) was the need to choose from a wide variety of possible specific models. Our proposed markers of acclimatization are not the only possible measures of acclimatization, which could also be parameterized in terms of a threshold for the heat effect in a linear threshold model or as a change in the slope of the exposure-response function, among other possibilities. For example, several studies have compared the MMT values across cities (Baccini et al. 2008, 2011; Curriero et al. 2002; McMichael et al. 2008) and over time using longer time series data (Honda et al. 2006). Our exploratory analyses suggest that such an approach would be even more limited in power for UHI decile groups within a single city, such as London, than the approaches we used. In addition, we preferred not to assume constant slopes in different UHI decile groups. Hence, we favor the “shifted spline” approach because it requires less-restrictive assumptions and makes fuller use of the data than most alternatives, and it is amenable to relatively straightforward statistical inference on the extent of acclimatization. Nonetheless, we recognize that acclimatization could result in other transformations in the shape of the temperature–mortality function.

When applied to London, our methods yielded relatively imprecise estimates of UHI acclimatization. For heat, this may in part be a result of the limited number of days in London with heat-related mortality. In addition, this may explain the contrast of our results with studies finding higher heat risks in areas more affected by UHI, which were all in cities with a higher proportion of heat-affected days (Goggins et al. 2012, 2013), as indicated by the MMTs found by Gasparrini et al. (2015). However, it was a surprise that there was not more power to discriminate between full and no acclimatization for cold, which accounts for a much larger overall burden of mortality in London. Here, the limitation seems to have been due to the less-steep slope for cold mortality compared with that for heat mortality (1.03/°C compared to 1.07/°C) which increases the difficulty in discriminating cold-mortality associations in localities with different UHIas.

## Conclusions

We have proposed and applied analytical methods that provide quantitative estimates of the degree of acclimatization to the heat- and cold-related mortality burdens associated with the UHI effect by comparing differences over area rather than changes over time. For London, our evidence suggests relatively full acclimatization to the UHI effect on summer heat–related mortality but less-clear evidence on the extent of acclimatization to the UHI effect for cold deaths. Evidence of the ability to acclimatize to the modest summer increments in temperatures related to the UHI for only London has limited relevance to policies to protect against future heat effects within cities experiencing climate change, but these methods could be applied to larger populations to inform such policies.

## Supplemental Material

(577 KB) PDF
