# Supplementary material for: Methods to Estimate Acclimatization to Urban Heat Island Effects on Heat- and Cold-Related Mortality
Source: Environ Health Perspect. 2016 Feb 9;124(7):1016–22. doi: 10.1289/ehp.1510109 (PMC4937865; doi:10.1289/ehp.1510109)
Supplement: (577 KB) PDF [file ehp.1510109.s001.acco.pdf]

Environ Health Perspect
DOI: 10.1289/ehp.1510109

Note to readers with disabilities: *EHP* strives to ensure that all journal content is accessible to all readers. However, some figures and Supplemental Material published in *EHP* articles may not conform to 508 standards due to the complexity of the information being presented. If you need assistance accessing journal content, please contact ehp508@niehs.nih.gov. Our staff will work with you to assess and meet your accessibility needs within 3 working days.

Supplemental Material

Methods to Estimate Acclimatization to the Urban Heat Island Effects on Heat- and Cold-Related Mortality

Ai Milojevic, Ben G. Armstrong, Antonio Gasparrini, Sylvia I. Bohnenstengel, Benjamin Barratt, and Paul Wilkinson

Table of Contents

Table S1. Heat- and cold-related relative risks (RR) at UHI anomalies (UHIa) of +0.5 and -0.5°C and observed interaction rate ratios (IRR) adjusted for socio-economic deprivation and those expected if there were no acclimatization.

Table S2. Heat- and cold-related relative risks (RR) for the least and the most deprived groups and observed interaction rate ratios (IRR) with and without adjustment for UHI anomaly (UHIa).

Table S3. Age-group specific heat- and cold-related relative risks (RR) at UHI anomalies (UHIa) of +0.5 and -0.5°C and observed interaction rate ratios (IRR) with and without adjustment for socio-economic deprivation and IRRs expected if there were no acclimatization.

Table S4. Heat- and cold-related relative risks (RR) at UHI anomalies (UHIa) of +0.5 and -0.5°C and observed interaction rate ratios (IRR) and those expected if there were no acclimatization, after adjusted for ambient pollution (O_3 and PM_{10}).

Table S5. Age-group specific heat- and cold-related relative risks (RR) at UHI anomalies (UHIa) of +0.5 and -0.5°C and observed interaction rate ratios (IRR) with and without adjustment for socio-economic deprivation and IRRs expected if there were no acclimatization [with shortened non-summer months, October - April].

Figure S1. The Wald z values for a range of candidate cutpoints defining hot and cold days (approach (i)). The values used in our analyses (6.4 and 22.3°C) are those with maximum z values.

Figure S2. Schematic illustrating how to estimate expected Interaction Rate Ratio (IRR) for heat. The same model as the main model was fit except: excluding an interaction term of temperature and UHI anomalies; and temperature effect modelled as a linear spline (segmented linear model) with knots at the minimum mortality temperature (18.6°C) and the higher cut-point (22.3°C). Expected IRR for heat is estimated as the slope in the spline above the highest knot.

Figure S3. Theoretical patterns of heat-mortality functions in 'Shifted splines' analysis under *no* acclimatization [A], where the curves for UHI anomalies (UHIa) of +0.5 ˚C (dashed line) and -0.5 ˚C (dotted line) are laterally displaced by +0.5 ˚C and -0.5 ˚C respectively from the London overall curve; and *full* acclimatization [B], where there is no displacement of curves for UHIa of +0.5 ˚C and -0.5 ˚C, which are therefore superimposed.

Figure S4. Temperature-mortality functions assuming acclimatization is neutral (γ=0.5) between full (γ=0) and none (γ=1) (left) and deviances of lateral displacement for values of γ in the range -0.5 to 1.5 ˚C (right) for summer heat (lags 0 to 1 days, June to August) [A] and winter cold (lags 0 to 13 days, September to May) [B], for those aged 75+ years only. Gray shading in the temperature mortality functions represent 95% CI. Deviances were calculated against maximum likelihood estimate (MLE). Likelihood ratio test (LRT) was applied for differences between deviances at γ =1 and γ =0.

Figure S5. Temperature-mortality functions assuming acclimatization is neutral (γ=0.5) between full (γ=0) and none (γ=1) (left) and deviances of lateral displacement for values of γ in the range -0.5 to 1.5 ˚C (right) for summer heat (lags 0 to 1 days, June to August) [A] and winter cold (lags 0 to 13 days, September to May) [B], after adjusted for O_3 and PM_{10}. Gray shading in the temperature mortality functions represent 95% CI. Deviances were calculated against maximum likelihood estimate (MLE). Likelihood ratio test (LRT) was applied for differences between deviances at γ =1 and γ =0.

Table S1. Heat- and cold-related relative risks (RR)[a] at UHI anomalies (UHIa)[b] of +0.5 and -0.5°C and observed interaction rate ratios (IRR)[c] adjusted for socio-economic deprivation and those expected if there were no acclimatization[d].

Exposure	UHIa[b] (°C)	Adjusted for deprivation		Expected IRR assuming *no* acclimatization[d]
		RR[a] (95%CI)	IRR[c] (95%CI)	
Heat	- 0.5	1.205 (1.155, 1.258)	1	1
	+ 0.5	1.188 (1.080, 1.306)	0.986 (0.879, 1.105)	1.070 (1.057, 1.082)
Cold	+ 0.5	1.152 (1.073, 1.237)	1	1
	- 0.5	1.150 (1.113, 1.187)	0.998 (0.916, 1.087)	1.030 (1.026, 1.034)

[a] RRs of mortality for heat and cold days with daily mean temperatures > 22.3 °C or < 6.4 °C (respectively) compared to days with daily mean temperatures ≥6.4 and ≤ 22.3 °C, with lag0–1 or lag0–13 (respectively) and adjustment for the day of the week, influenza counts and socio-economic deprivation.
[b] UHI anomaly was defined as the average of excess daily mean temperature (°C) at 1km grid compared to the London overall temperature.
[c] Ratios of the RR for heat in UHIa +0.5 vs. -0.5 °C, or of the RR for cold in UHIa -0.5 vs. 0.5 °C.
[d] Expected IRRs are generated by modelling the association between mortality and daily mean temperature for London as a whole using a linear spline with knots at 18.6 °C (the minimum mortality temperature) and at 22.3 °C (for heat) or at 6.4 °C and 18.6 °C (for cold), with each IRR representing the risk of mortality with a 1°C increase in daily mean temperature > 22.3 °C or < 6.4 °C for heat and cold, respectively. Expected IRR is estimated by time-series analysis using time-invariant socio-economic deprivation variable, as such not confounded by deprivation.

Table S2. Heat- and cold-related relative risks (RR)[a] for the least and the most deprived groups[b] and observed interaction rate ratios (IRR)[c] with and without adjustment for UHI anomaly (UHIa)[d].

Exposure	Deprivation group[b]	Unadjusted		Adjusted for UHIa[d]	
		RR[a] (95%CI)	IRR[c] (95%CI)	RR[a] (95%CI)	IRR[c] (95%CI)
Heat	The least deprived	1.202 (1.161, 1.244)	1	1.205 (1.155, 1.258)	1
	The most deprived	1.213 (1.167, 1.261)	1.010 (0.949, 1.074)	1.235 (1.070, 1.424)	1.024 (0.901, 1.164)
Cold	The most deprived	1.120 (1.088, 1.154)	1	1.117 (1.004, 1.244)	1
	The least deprived	1.150 (1.121, 1.180)	1.027 (0.980, 1.076)	1.150 (1.113, 1.187)	1.029 (0.935, 1.133)

[a] RRs of mortality for heat and cold days with daily mean temperatures > 22.3 °C or < 6.4 °C (respectively) compared to days with daily mean temperatures ≥6.4 and ≤ 22.3 °C, with lag0–1 or lag0–13 (respectively) and adjustment for the day of the week and influenza counts with/without socio-economic deprivation.
[b] Deprivation groups were divided into decile groups by English Index of Multiple Deprivation excluding the health and disability domains and the living environment domain, from which only the least and the most deprived groups were compared here.
[c] Ratio of the RR for the most deprived group against the least deprived group.
[d] UHI anomaly was defined as the average of excess daily mean temperature (°C) at 1km grid compared to the London overall temperature.

Table S3. Age-group specific heat- and cold-related relative risks (RR)[a] at UHI anomalies (UHIa)[b] of +0.5 and -0.5°C and observed interaction rate ratios (IRR)[c] with and without adjustment for socio-economic deprivation[d] and IRRs expected if there were no acclimatization[e].

Age / exposure	UHIa[b] (°C)	Unadjusted		Adjusted for deprivation[d]		Expected IRR assuming *no* acclimatization[e]
		RR[a] (95%CI)	IRR[c] (95%CI)	RR[a] (95%CI)	IRR[c] (95%CI)	
All age						
Heat	- 0.5	1.203 (1.154, 1.255)	1	1.205 (1.155, 1.258)	1	1
	+ 0.5	1.208 (1.176, 1.241)	1.004 (0.950, 1.061)	1.188 (1.080, 1.306)	0.986 (0.879, 1.105)	1.070 (1.057, 1.082)
Cold	+ 0.5	1.129 (1.106, 1.152)	1	1.152 (1.073, 1.237)	1	1
	- 0.5	1.152 (1.116, 1.189)	1.020 (0.978, 1.063)	1.150 (1.113, 1.187)	0.998 (0.916, 1.087)	1.030 (1.025, 1.034)
Age 0-64 yrs						
Heat	- 0.5	1.124 (1.015, 1.244)	1	1.104 (0.991, 1.229)	1	1
	+ 0.5	1.142 (1.080, 1.207)	1.016 (0.896, 1.152)	1.278 (1.025, 1.594)	1.158 (0.877, 1.529)	1.036 (1.009, 1.064)
Cold	+ 0.5	1.069 (1.023, 1.117)	1	1.088 (0.915, 1.294)	1	1
	- 0.5	1.072 (0.990, 1.161)	1.003 (0.908, 1.107)	1.070 (0.984, 1.163)	0.983 (0.792, 1.220)	1.016 (1.005, 1.027)
Age 65-74 yrs						
Heat	- 0.5	1.108 (1.002, 1.225)	1	1.121 (1.011, 1.243)	1	1
	+ 0.5	1.133 (1.066, 1.204)	1.023 (0.899, 1.163)	1.020 (0.817, 1.273)	0.910 (0.695, 1.191)	1.047 (1.018, 1.077)
Cold	+ 0.5	1.096 (1.048, 1.146)	1	1.237 (1.051, 1.455)	1	1
	- 0.5	1.144 (1.063, 1.231)	1.044 (0.950, 1.147)	1.129 (1.046, 1.217)	0.913 (0.749, 1.112)	1.039 (1.028, 1.049)
Age 75+ yrs						
Heat	- 0.5	1.244 (1.181, 1.311)	1	1.248 (1.184, 1.316)	1	1
	+ 0.5	1.266 (1.221, 1.312)	1.017 (0.948, 1.091)	1.210 (1.074, 1.365)	0.969 (0.842, 1.117)	1.086 (1.070, 1.102)
Cold	+ 0.5	1.161 (1.130, 1.192)	1	1.141 (1.044, 1.247)	1	1
	- 0.5	1.173 (1.128, 1.219)	1.011 (0.959, 1.065)	1.174 (1.129, 1.222)	1.029 (0.926, 1.144)	1.031 (1.025, 1.037)

[a] RRs of mortality for heat and cold days with daily mean temperatures > 22.3 °C or < 6.4 °C (respectively) compared to days with daily mean temperatures ≥6.4 and ≤ 22.3 °C, with lag0–1 or lag0–13 (respectively) and adjustment for the day of the week, influenza counts with/without socio-economic deprivation.

[b] UHI anomaly was defined as the average of excess daily mean temperature (°C) at 1km grid compared to the London overall temperature.

[c] Ratios of the RR for heat in UHIa +0.5 vs. -0.5 °C, or of the RR for cold in UHIa -0.5 vs. 0.5 °C.

[d] Deprivation was adjusted by entering an average of reconstructed EIMD scores by UHI decile groups as a further interaction terms with heat or cold in the model [3].

[e] Expected IRRs are generated by modelling the association between mortality and daily mean temperature for London as a whole using a linear spline with knots at 18.6 °C (the minimum mortality temperature) and at 22.3 °C (for heat) or at 6.4 °C and 18.6 °C (for cold), with each IRR representing the risk of mortality with a 1°C increase in daily mean temperature > 22.3 °C or < 6.4 °C for heat and cold, respectively.

Table S4. Heat- and cold-related relative risks (RR)[a] at UHI anomalies (UHIa)[b] of +0.5 and -0.5°C and observed interaction rate ratios (IRR)[c] and those expected if there were no acclimatization[d], after adjusted for ambient pollution (O_3 and PM_{10})[e].

Exposure	UHIa[b] (°C)	Adjusted for ambient pollution[e]		Expected IRR assuming *no* acclimatization[d]
		RR[a] (95%CI)	IRR[c] (95%CI)	
Heat	- 0.5	1.116 (1.067, 1.167)	1	1
	+ 0.5	1.120 (1.087, 1.155)	1.004 (0.950, 1.061)	1.059 (1.046, 1.073)
Cold	+ 0.5	1.136 (1.112, 1.159)	1	1
	- 0.5	1.158 (1.122, 1.196)	1.020 (0.979, 1.063)	1.030 (1.026, 1.035)

[a] RRs of mortality for heat and cold days with daily mean temperatures > 22.3 °C or < 6.4 °C (respectively) compared to days with daily mean temperatures ≥6.4 and ≤ 22.3 °C, with lag0–1 or lag0–13 (respectively) and adjustment for the day of the week, influenza counts with/without socio-economic deprivation.

[b] UHI anomaly was defined as the average of excess daily mean temperature (°C) at 1km grid compared to the London overall temperature.

[c] Ratios of the RR for heat in UHIa +0.5 vs. -0.5 °C, or of the RR for cold in UHIa -0.5 vs. 0.5 °C.

[d] Expected IRRs are generated by modelling the association between mortality and daily mean temperature for London as a whole using a linear spline with knots at 18.6 °C (the minimum mortality temperature) and at 22.3 °C (for heat) or at 6.4 °C and 18.6 °C (for cold), with each IRR representing the risk of mortality with a 1°C increase in daily mean temperature > 22.3 °C or < 6.4 °C for heat and cold, respectively.

[e] Ambient pollution was adjusted by including daily maximum of 8 hours running mean of O_3 and daily mean of PM_{10} as a whole in London in the model as linear terms.

Table S5. Age-group specific heat- and cold-related relative risks (RR)[a] at UHI anomalies (UHIa)[b] of +0.5 and -0.5°C and observed interaction rate ratios (IRR)[c] with and without adjustment for socio-economic deprivation[d] and IRRs expected if there were no acclimatization[e] [with shortened non-summer months, October - April].

Age / exposure	UHIa[b] (°C)	Unadjusted		Adjusted for deprivation[d]		Expected IRR assuming *no* acclimatization[e]
		RR[a] (95%CI)	IRR[c] (95%CI)	RR[a] (95%CI)	IRR[c] (95%CI)	
All age						
Cold	+ 0.5	1.130 (1.107, 1.153)	1	1.154 (1.074, 1.239)	1	1
	- 0.5	1.152 (1.116, 1.189)	1.020 (0.978, 1.063)	1.149(1.113, 1.187)	0.996 (0.914, 1.086)	1.029 (1.024, 1.034)
Age 0-64 yrs						
Cold	+ 0.5	1.070 (1.024, 1.118)	1	1.093 (0.919, 1.300)	1	1
	- 0.5	1.075 (0.992, 1.164)	1.005 (0.910, 1.109)	1.071 (0.985, 1.165)	0.980 (0.790, 1.217)	1.015 (1.004, 1.026)
Age 65-74 yrs						
Cold	+ 0.5	1.097 (1.049, 1.147)	1	1.242 (1.056, 1.462)	1	1
	- 0.5	1.144 (1.062, 1.231)	1.043 (0.949, 1.146)	1.128 (1.045, 1.217)	0.908 (0.833, 1.024)	1.037 (1.026, 1.048)
Age 75+ yrs						
Cold	+ 0.5	1.161(1.131, 1.193)	1	1.140 (1.043, 1.246)	1	1
	- 0.5	1.172 (1.128, 1.219)	1.009 (0.958, 1.063)	1.174 (1.129, 1.221)	1.030 (0.927, 1.144)	1.030 (1.024, 1.036)

[a] RRs of mortality for heat and cold days with daily mean temperatures > 22.3 °C or < 6.4 °C (respectively) compared to days with daily mean temperatures ≥6.4 and ≤ 22.3 °C, with lag0–1 or lag0–13 (respectively) and adjustment for the day of the week, influenza counts with/without socio-economic deprivation.
[b] UHI anomaly was defined as the average of excess daily mean temperature (°C) at 1km grid compared to the London overall temperature.
[c] Ratios of the RR for heat in UHIa +0.5 vs. -0.5 °C, or of the RR for cold in UHIa -0.5 vs. 0.5 °C.
[d] Deprivation was adjusted by entering an average of reconstructed EIMD scores by UHI decile groups as a further interaction terms with heat or cold in the model [3].
[e] Expected IRRs are generated by modelling the association between mortality and daily mean temperature for London as a whole using a linear spline with knots at 18.6 °C (the minimum mortality temperature) and at 22.3 °C (for heat) or at 6.4 °C and 18.6 °C (for cold), with each IRR representing the risk of mortality with a 1°C increase in daily mean temperature > 22.3 °C or < 6.4 °C for heat and cold, respectively.

Cut-point for cold days

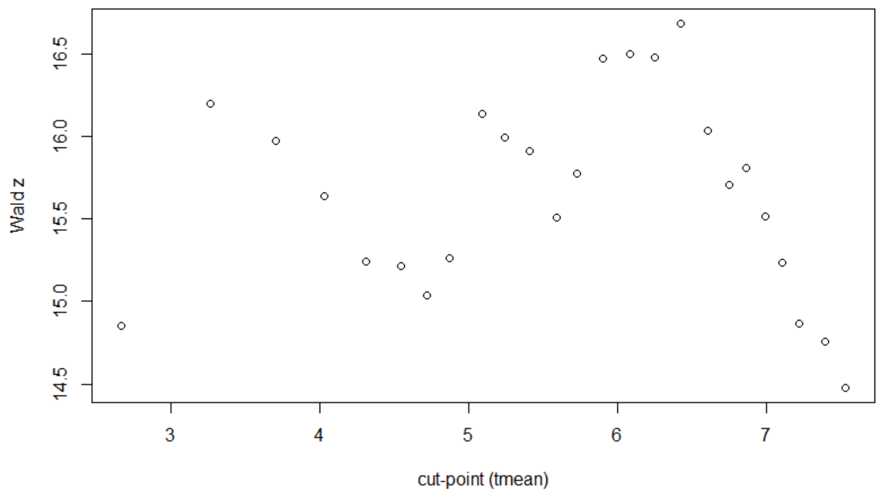

Cut-point for hot days

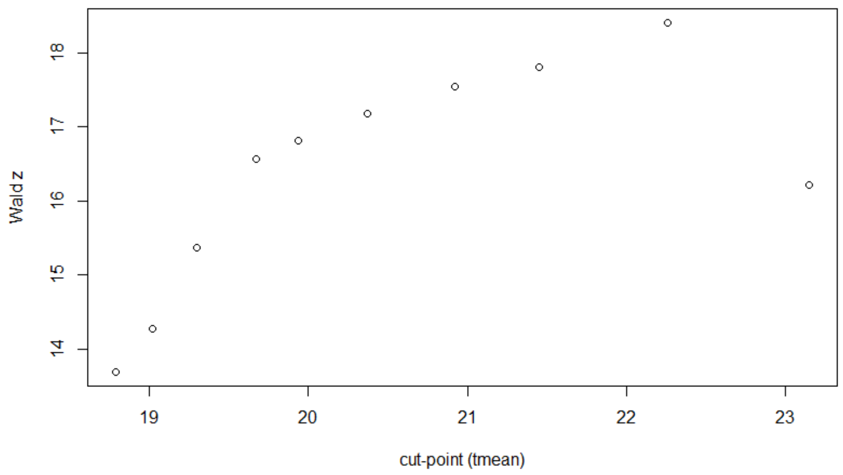

Figure S1. The Wald z values for a range of candidate cutpoints defining hot and cold days (approach (i)). The values used in our analyses (6.4 and 22.3°C) are those with maximum z values.

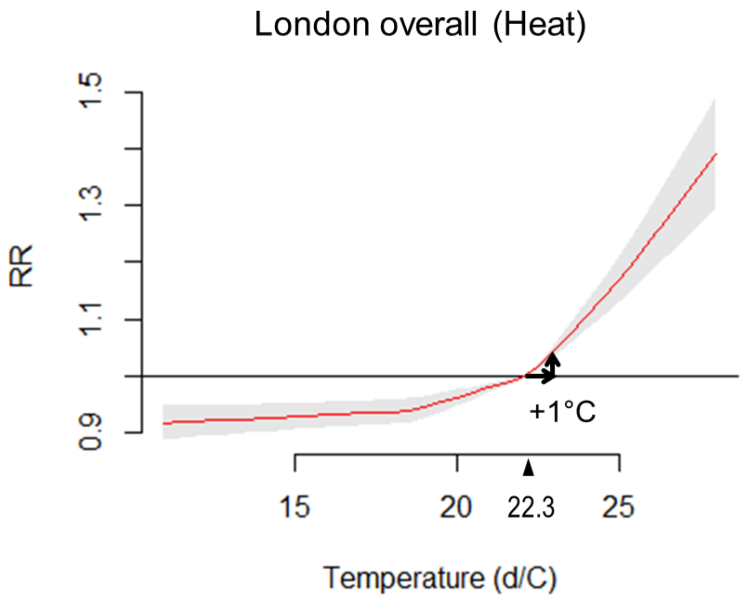

Figure S2. Schematic illustrating how to estimate expected Interaction Rate Ratio (IRR) for heat. The same model as the main model was fit except: excluding an interaction term of temperature and UHI anomalies; and temperature effect modelled as a linear spline (segmented linear model) with knots at the minimum mortality temperature (18.6°C) and the higher cut-point (22.3°C). Expected IRR for heat is estimated as the slope in the spline above the highest knot.

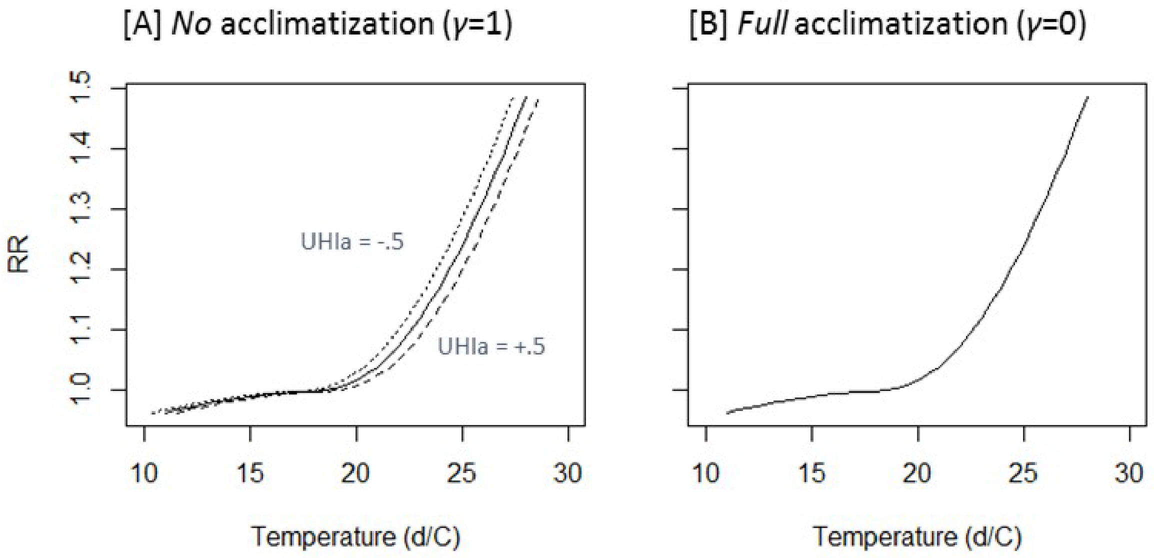

Figure S3. Theoretical patterns of heat-mortality functions in 'Shifted splines' analysis under *no* acclimatization [A], where the curves for UHI anomalies (UHIa) of +0.5 ˚C (dashed line) and -0.5 ˚C (dotted line) are laterally displaced by +0.5 ˚C and -0.5 ˚C respectively from the London overall curve; and *full* acclimatization [B], where there is no displacement of curves for UHIa of +0.5 ˚C and -0.5 ˚C, which are therefore superimposed.

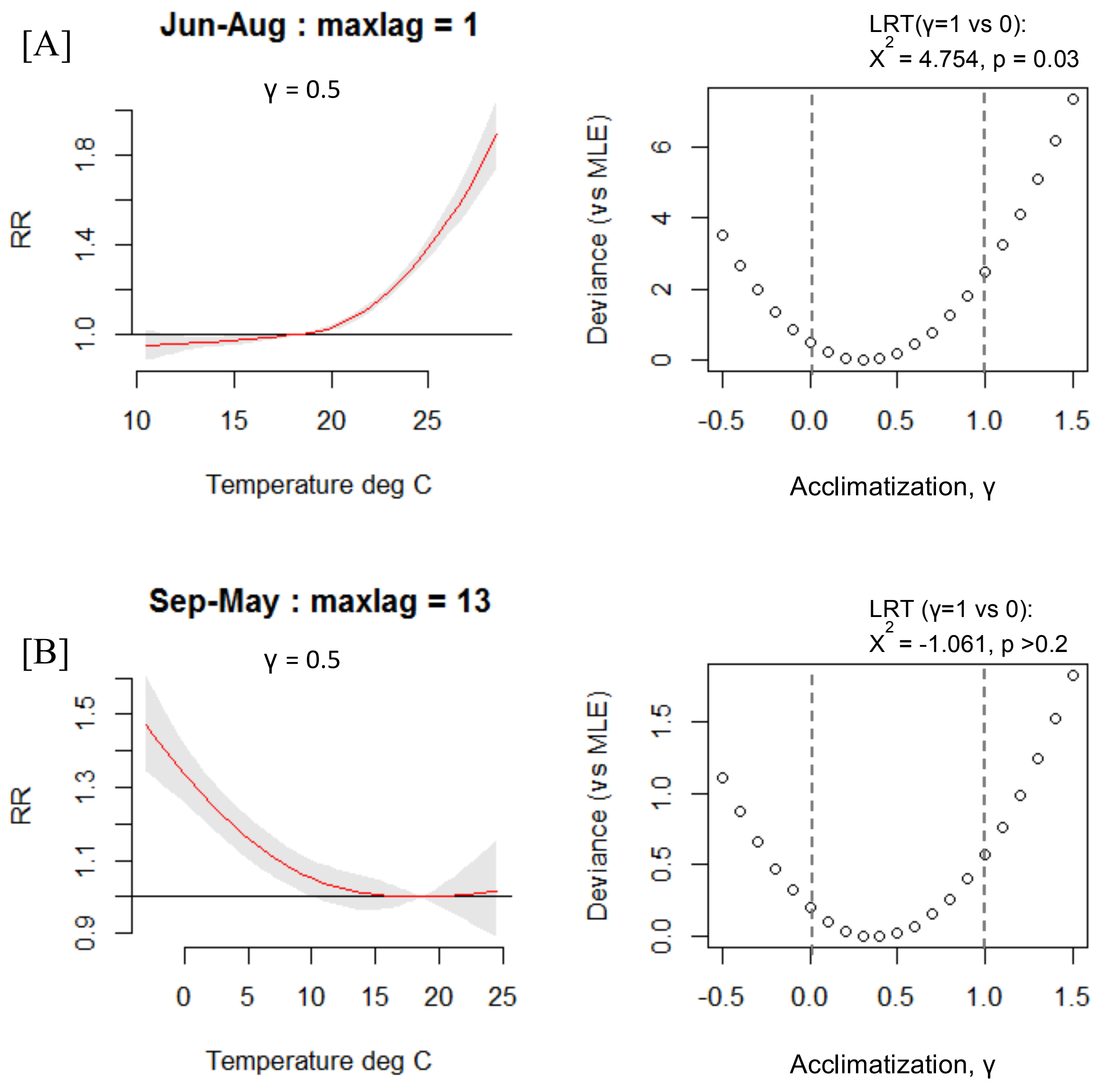

Figure S4. Temperature-mortality functions assuming acclimatization is neutral (γ=0.5) between full (γ=0) and none (γ=1) (left) and deviances of lateral displacement for values of γ in the range -0.5 to 1.5 ˚C (right) for summer heat (lags 0 to 1 days, June to August) [A] and winter cold (lags 0 to 13 days, September to May) [B], for those aged 75+ years only. Gray shading in the temperature mortality functions represent 95% CI. Deviances were calculated against maximum likelihood estimate (MLE). Likelihood ratio test (LRT) was applied for differences between deviances at γ =1 and γ =0.

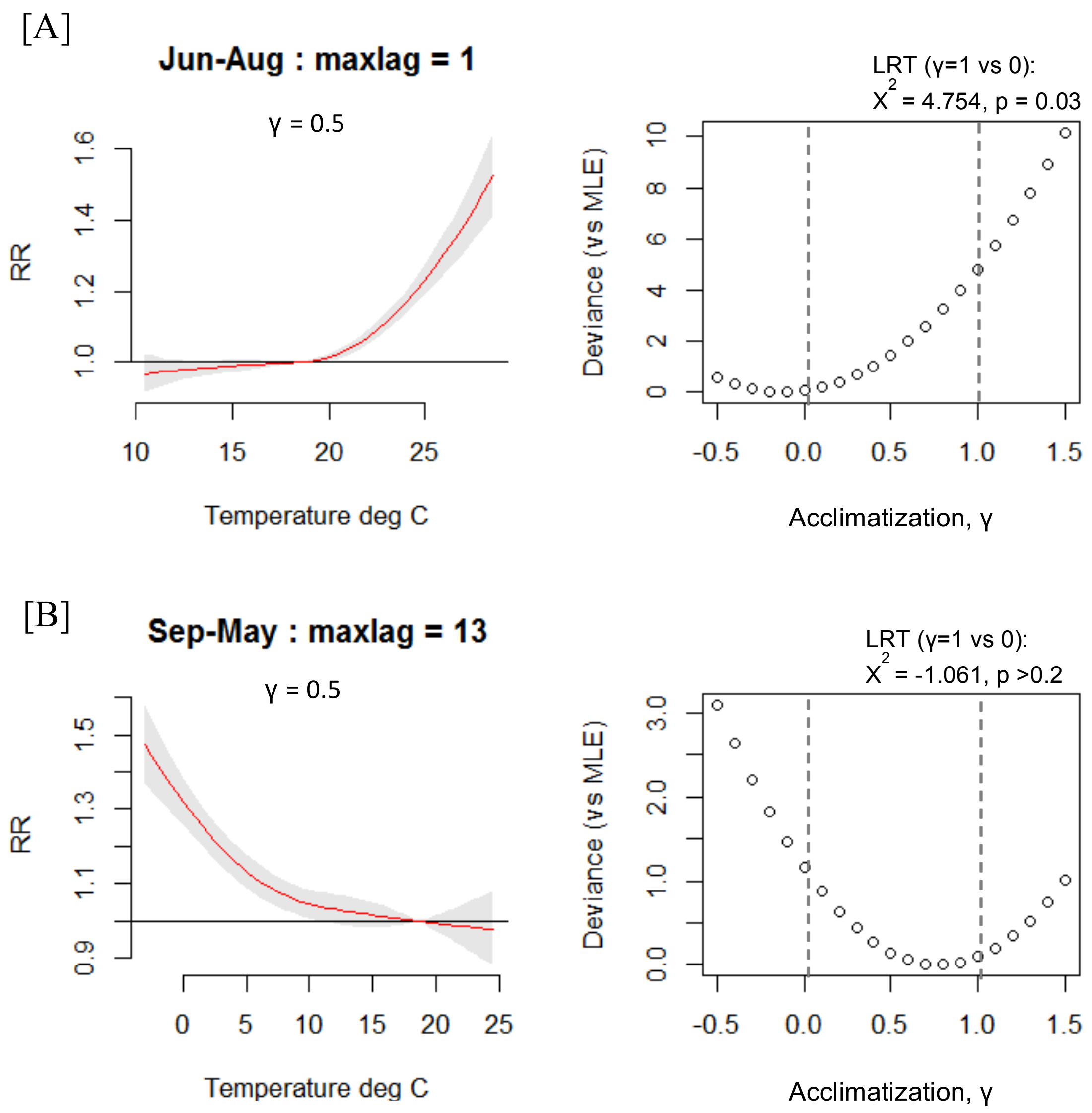

Figure S5. Temperature-mortality functions assuming acclimatization is neutral (γ=0.5) between full (γ=0) and none (γ=1) (left) and deviances of lateral displacement for values of γ in the range -0.5 to 1.5 ˚C (right) for summer heat (lags 0 to 1 days, June to August) [A] and winter cold (lags 0 to 13 days, September to May) [B], after adjusted for O_3 and PM_{10}. Gray shading in the temperature mortality functions represent 95% CI. Deviances were calculated against maximum likelihood estimate (MLE). Likelihood ratio test (LRT) was applied for differences between deviances at γ =1 and γ =0.
